# Suicidal attempts and ideations in Kenyan adults with psychotic disorders: An observational study of frequency and associated risk factors

**DOI:** 10.3389/fpsyt.2022.1085201

**Published:** 2023-01-19

**Authors:** Linnet Ongeri, Symon M. Kariuki, Miriam Nyawira, Chris Schubart, Joeri K. Tijdink, Charles R. J. C. Newton, Brenda W. J. H. Penninx

**Affiliations:** ^1^Centre for Clinical Research, Kenya Medical Research Institute, Nairobi, Kenya; ^2^Department of Public Health, Pwani University, Kilifi, Kenya; ^3^Neuroscience Unit, Kenya Medical Research Institute-Wellcome Trust Research Program, Kilifi, Kenya; ^4^Department of Psychiatry, University of Oxford, Oxford, United Kingdom; ^5^Tergooi Medical Centre, Hilversum, Netherlands; ^6^Department of Ethics, Law and Humanities, Amsterdam UMC, Vrije Universiteit Amsterdam, Amsterdam, Netherlands; ^7^Department of Philosophy, Faculty of Humanities, Vrije Universiteit Amsterdam, Amsterdam, Netherlands; ^8^Department of Psychiatry, Amsterdam Public Health, Amsterdam UMC, Vrije Universiteit Amsterdam, Amsterdam, Netherlands

**Keywords:** suicidal attempts, suicidal ideation, schizophrenia, Kenya, sub-Saharan Africa, low and middle income countries, risk factors, stigma

## Abstract

**Background:**

Psychotic disorders increase the risk for premature mortality with up to 40% of this mortality attributable to suicide. Although suicidal ideation (SI) and suicidal behavior (SB) are high in persons with psychotic disorders in sub-Saharan Africa, there is limited data on the risk of suicide and associated factors among persons with psychotic disorders.

**Methods:**

We assessed SI and SB in persons with psychotic disorders, drawn from a large case-control study examining the genetics of psychotic disorders in a Kenyan population. Participants with psychotic disorders were identified using a clinical review of records, and the diagnosis was confirmed with the Mini-International Neuropsychiatric Interview (MINI). We conducted bivariate and multivariate logistic (for binary suicide outcomes) or linear regression (for suicide risk score) analysis for each of the suicide variables, with demographic and clinical variables as determinants.

**Results:**

Out of 619 participants, any current SI or lifetime suicidal attempts was reported by 203 (32.8%) with psychotic disorders, of which 181 (29.2%) had a lifetime suicidal attempt, 60 (9.7%) had SI in the past month, and 38 (20.9%) had both. Family history of suicidality was significantly associated with an increased risk of suicidality across all the following four outcomes: SI [OR = 2.56 (95% CI: 1.34–4.88)], suicidal attempts [OR = 2.01 (95% CI: 1.31–3.06)], SI and SB [OR = 2.00 (95% CI: 1.31–3.04)], and suicide risk score [beta coefficient = 7.04 (2.72; 11.36), *p* = 0.001]. Compared to persons aged <25 years, there were reduced odds for SI for persons aged ≥ 25 years [OR = 0.30 (95% CI: 0.14–0.62)] and ≥ 45 years [OR = 0.32 (95% CI: 0.12–0.89)]. The number of negative life events experienced increased the risk of SI and SB [OR = 2.91 (95% CI: 1.43–5.94)] for 4 or more life events. Higher negative symptoms were associated with more suicidal attempts [OR = 2.02 (95%CI: 1.15–3.54)]. Unemployment was also associated with an increased risk for suicidal attempts [OR = 1.58 (95%CI: 1.08–2.33)] and SI and SB [OR = 1.68 (95% CI: 1.15–2.46)].

**Conclusion:**

Suicidal ideation and SB are common in persons with psychotic disorders in this African setting and are associated with sociodemographic factors, such as young age and unemployment, and clinical factors, such as family history of suicidality. Interventions targeted at the community (e.g., economic empowerment) or at increasing access to care and treatment for persons with psychotic disorders may reduce the risk of suicide in this vulnerable population group.

## Introduction

Psychotic disorders such as schizophrenia are not only disabling conditions but also are an important cause of premature mortality, with an increased mortality risk of up to 7–10% (1, 2). Of the deaths related to psychotic disorders, 40% are thought to be due to suicide (3). In fact, approximately 60% of people with psychotic disorders have suicidal behavior (SB) at one point in their lifetime, and their risk of dying by suicide is 8.5 times higher than that in the general population (4, 5). There are few studies of suicidal ideation (SI) and suicidal behavior (SB) in low- and middle-income countries (LMIC), including sub-Saharan Africa, where rates of mental disorders and SB are high (6) and where there are limited mental health services, leading to a large mental health treatment gap (7).

The greatest risk for suicide in psychotic disorders is in the first year after the first admission, when the risk of dying by suicide is 12 times higher in the general population, representing two-thirds of all psychosis-related suicides (8). This greater risk of suicide in the early phase of a psychotic disorder may be attributable to the preservation of some insight and thus awareness of current or future limitations due to the psychotic illness (9). Other clinical, psychotic-illness-related, and demographic factors have been implicated in the greater risk of suicide. For example, young age at onset and male sex are known to increase the risk of suicide, although there have been conflicting results about the age of onset that could be ascribed to differences in study design (10). The risk is also increased by a lack of social support and traumatic experiences, which may have a long-term impact on neurochemistry and brain structure (11). For psychotic-illness factors, the severity of diseases, e.g., many positive symptoms especially hallucinations and low functioning, has been implicated in elevated risk (12). Comorbid mood disorders in persons with psychosis also increase the risk of suicide (13). While some studies found that negative symptoms in patients with psychotic disorders are protective from suicidal behavior (12), others have reported SI and SB to be up to 8-fold greater in persons with prodromal negative symptoms compared to controls (14). Comorbid substance use disorders are common in persons with psychotic disorders (15), and these are strongly linked to increased suicide risk (16). However, these data are based on studies of suicide and psychosis in high-income countries, but few are from low- and middle-income countries, and almost no studies have been conducted in sub-Saharan Africa.

Recent epidemiological studies in Kenya have found high estimates for suicidal attempts (17%) (17) and psychotic symptoms (16.7%) (18), which could suggest that more investigations are needed to establish whether there is a close relationship between the two and to characterize how these factors are influencing each other. There are, however, no well-powered or properly designed studies on the relationship between the two in the Kenyan setting. Furthermore, associated factors of psychosis, such as negative life events, psychological and medical comorbidities, and the symptomatology of psychosis, may be important antecedents for suicidality in LMIC as they are in other settings worldwide. However, these have also not been systematically studied in sub-Saharan Africa.

We aim to determine the sociodemographic and clinical factors associated with recent SI and lifetime suicidal attempts in a Kenyan sample of patients with psychotic disorders. Identified factors can improve the formulation of the suicide risk and provide knowledge on how to apply suicide prevention measures applicable in the sub-Saharan region by clinicians evaluating suicide risk in patients with psychotic disorders.

## Materials and methods

### Sample and setting

**FIGURE 1:**
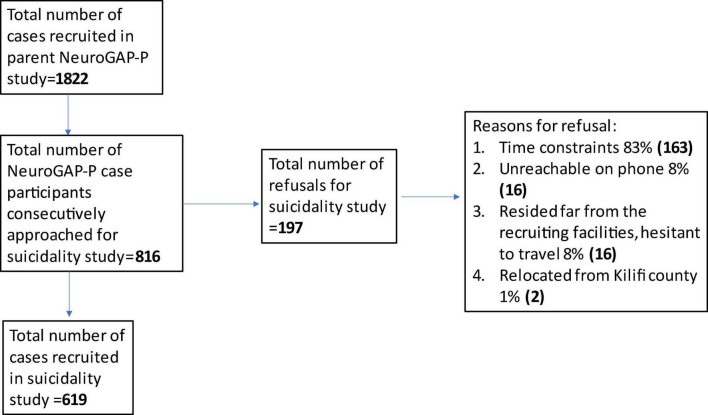
Flowchart on participant recruitment.

In this study, we used a cross-sectional study design to assess suicidality in persons with psychotic disorders, who were drawn from a large multisite and multiregional case-control study examining the genetics of psychotic disorders in African populations: the Neuropsychiatric Genetics of African Populations-Psychosis (NeuroGAP-P) study (19). The NeuroGAP-P study included participants from 4 sub-Saharan African countries (i.e., Kenya, Uganda, South Africa, and Ethiopia). Cases in the parent case-control genetic study were defined as outpatients with schizophrenia or bipolar 1 disorder with psychosis symptoms; both were grouped as psychotic disorders because of significant clinical and genetic overlap between the two conditions (20). Our targeted sample size for cases was 550 patients. For our sub-study, we only recruited from the coastal Kenya study site; from an overall sampling frame of 3,155 participants, 1,153 (57.7%) had psychotic disorders (see flowchart Figure 1).

Potential case participants for the NeuroGAP-P parent study were identified by clinical staff through the review of medical records. A trained research assistant clinician then consecutively approached the prospective participant in a private space at the outpatient mental health units and read out the consent document information sheet. The University of California, San Diego Brief Assessment of Capacity to Consent (UBACC) tool was used during consenting to ensure sufficient autonomy and assess capacity to consent. Potential participants who scored less than 14.5 out of a full score of 20 were excluded after a maximum of four trials. Participants who achieved the cutoff score and who expressed interest in participation were invited to provide written informed consent.

Study participants in the Coastal Kenya site were recruited from four large government-run health facilities [Kilifi County Hospital (KCH), Malindi Sub-County Hospital (MCH), Coast General County Hospital (CGCH), and Port Reitz Hospital (PRH) in Mombasa]. All four facilities have mental health units that offer outpatient mental health services. In addition to the outpatient units, only Port Reitz Hospital has an inpatient admitting unit with a bed capacity of 72. The other three facilities refer patients to Port Reitz for admission to the inpatient unit. All the facilities are staffed by a clinical team consisting of clinical officers specializing in psychiatry, psychiatric nurses, and clinical psychologists. At the time of data collection, one government psychiatrist who worked between Mombasa County Hospital and Port Reitz Hospital served the entire coastal region.

Inclusion criteria for the parent NeuroGAP-P study for cases included an outpatient with a diagnosis of a psychotic disorder (schizophrenia, schizophreniform disorder, schizoaffective disorder, brief psychotic disorder, substance-induced psychotic disorder, and psychotic disorder not otherwise specified) or bipolar 1 disorder with psychotic features. Psychotic disorder diagnosis was made based on module K (psychotic disorders and mood disorders with psychotic features) of the Mini-International Neuropsychiatry Interview (MINI) version 7.0.2 for Diagnostic Statistical Manual Version 5 (DSM-5) criteria (21). The module codes psychotic disorders into 4 categories, namely, current and lifetime mood disorders with psychotic features, and current and lifetime psychotic disorders. Any of the 4 categories fulfilled eligibility for recruitment as a case in the study. All participants had to be able to understand either English or Kiswahili (the local language), be aged 18 years and above, and provide written informed consent.

Following the completion of the parent study procedures (19), participants at the Kenyan Coast study site were informed about the suicidality sub-study [suicidality in psychosis study (SIPs)] by the NeuroGAP-P research assistants. Participants interested in hearing more about the suicidality study were introduced to the suicidality research assistants, and study information was then provided. Participants were consecutively recruited into the SIPs sub-study only after providing written informed consent. Study participants were also retrospectively selected from the NeuroGAP-P parent study records. In this case, research assistants only did callbacks using the contact information provided by participants who had consented to call back for subsequent future research, as recorded in consent forms. Callbacks were done consecutively from the list provided as potential participants. The UBACC was not repeated for the suicidality sub-study.

For this suicidality study, patients were eligible for inclusion if they (i) had been recruited for the parent NeuroGAP-P study, (ii) had consented to be contacted for follow-up studies, and (iii) were willing and able to sign a written informed consent form for the nested suicidal behavior sub-study. Potential cases were excluded if they had severe and intrusive levels of psychotic symptoms at the time of consenting if they were intoxicated or experiencing withdrawal from alcohol or substance abuse, if they were inpatients with a current psychiatric hospitalization, or if they had been involuntarily detained at the time of consenting.

### Measures and procedures for the suicidality sub-study

Data collection for this nested sub-study was conducted between 18 November 2019 and 19 November 2021.

#### Suicidality outcome measure

Assessment of SI, current and lifetime suicidal attempts, and suicide risk score in the present study was done with MINI version 7.0.2 for DSM 5 module B (22). This tool has been validated in a number of studies (23). Current SI was assessed using question 3B, “In the past 1 month did you think (even momentarily) about harming or of hurting or of injuring yourself; with at least some intent or awareness that you might die as a result or think about suicide (i.e., about killing yourself)?” Lifetime suicidal attempt was assessed using question B18, “In your lifetime, did you ever make a suicide attempt (try to kill yourself)?” The overall suicide risk score was measured using the sum of the weighted scale for the 16 questions in the suicidality module. The categorization of suicidality risk into low, moderate, and high risk was measured using the scale of module for an overall score of 1–8 as low risk, 9–16 as moderate risk, and ≥17 points as high suicidality risk.

#### Baseline demographic characteristics

A sociodemographic schedule was used across our nested SB sub-study to record participants’ demographic information such as age, sex, level of education, marital status, employment status, level of household income, and family history of SB.

#### Clinical characteristics

Measured features of psychosis included any past or current presentation of hallucinations, delusions, disordered thinking and behavior, and negative symptoms measured using the MINI version 7 for DSM-5 module K.

Other clinical data were collected in the parent NeuroGAP-P study and merged with our suicidality and sociodemographic data using the unique participant identifier. The comorbidity of major depressive disorders was assessed using the MINI version 7, Module A: Major Depressive Episode. The comorbidity of bipolar mood disorders (manic and hypomanic episodes) was assessed using MINI version 7, Module K. Life events, composed of natural disasters, fires or explosions, transport and other serious accidents, exposure to toxic substances, assault and combat, or exposure to war, among others, were assessed using the Life Event Checklist (LEC 5), which assesses for lifetime exposure to traumatic events (24). The presence of comorbid chronic conditions ranging from chronic pain, hypertension, and diabetes was assessed using the Composite International Diagnostic Interview (CIDI) chronic conditions screener (25). Comorbid substance use disorder was assessed using the WHO Alcohol, Smoking, and Substance Involvement Screening Test (ASSIST) (26).

### Statistical analyses

Data were analyzed using Stata version 17 (27). We constructed 3 separate dichotomous dependent variables denoting the presence or absence of recent SI (in the past 30 days), current or lifetime suicidal attempts, and an integrative SI and SB measure, which was defined as SI and/or current or lifetime suicidal attempts. We conducted bivariate logistic regression analysis for each of the dependent (outcome) suicide variables, with demographic and clinical variables. We then built multivariate logistic regression models using independent variables with a *p*-value of ≤0.25 in the bivariate analysis for each suicide-dependent variable (any SI, suicidal attempts, and SI and SB). In addition, the Akaike Information Criteria (AIC) values associated with the models were considered in the fitness of the model, but the *p*-value criterion was still used to preselect variables for inclusion in a multivariate model. We repeated these regression analyses for the overall suicide risk score as a continuous dependent variable, using a generalized linear model with specifications for logarithmic scales because the risk score did not follow a Gaussian distribution. Population attributable risk percentage (PAR) (the proportion of suicidality in the population (exposed and unexposed) that would be eliminated if the exposure was avoided) was estimated for each risk factor that was significant in the multivariate models and can be addressed through preventative and therapeutic interventions. *T*-test was used to compare means for normally distributed continuous variables, the Mann-Whitney test for normally distributed non-continuous variables, and the Pearson chi-squared test for categorical variables. For categorical variables with non-frequent outcomes, we used Fisher’s exact test.

We report associations between independent variables (sociodemographic and clinical factors) and dependent variables (suicidality outcomes) as unadjusted and adjusted odds ratios (ORs) with their 95% confidence intervals. All adjusted analyses were considered significant at the 5% level, *p* < 0.05. The multicollinearity for the variables included in the multivariate model was checked using the variance inflation (VIF) matrix to ensure that none of them was substantially large, i.e., a VIF less than 10. All variables had a modest VIF of approximately 1, ruling out any concerns of multicollinearity among them (28) (Supplementary Table 5).

### Ethical consideration

All participants in the parent study and nested suicidality sub-study provided written informed consent to participate in the studies. The parent study and the nested sub-study were approved by the Kenya Medical Research Institute Scientific and Ethics Review Unit (SERU:3575, SERU:3916), respectively.

## Results

### General description of study participants

Out of a sampling frame of 1,822 cases of patients with psychotic disorders recruited in the parent NeuroGAP-P study at the Kenya Coastal region site, we recruited 619 consecutive participants into the suicidality sub-study following a response rate of 75% (Figure 1). There were 391 (63.2%) men, 193 (31.2%) married, and 257 (41.5%) unemployed (Table 1). Some clinical history factors differed by sex, e.g., more men than women reported substance use (83.1% vs. 34.7%, *p* < 0.001), while women reported more chronic physical illnesses than men (65.6% vs. 47.6%, *p* < 0.001). Comparison analysis of the participants in the suicidality study with the case participants not selected from the parent project (1,180) found more men (*p* = 0.004) and more negative symptoms (*p* = 0.041) in our suicidality study, but no differences in age (*p* = 0.116) and auditory hallucinations (*p* = 0.106) (Supplementary Table 1). The most reported positive psychotic symptom was auditory hallucinations (77%), followed by delusions of control (75%), while the lifetime presence of negative symptoms was reported by 81% (Supplementary Table 2). The majority of the study participants had psychotic disorders with comorbid substance use and bipolar disorder (38.2%), followed by psychotic disorders with comorbid bipolar disorder (24.8%). Only 4.5% of the participants had a psychotic disorder without other mental disorder comorbidities assessed (Supplementary Table 3). To further explore whether mental health comorbidity may have affected our results, we compared the distribution of SI and SB between those with comorbidity (*n* = 591) and those without any comorbidity (*n* = 28) and found that the difference was not statistically significant (33% vs. 25%, *p* = 0.369).

**TABLE 1:** Sociodemographic and clinical characteristics by gender of the study sample.

Characteristics	Overall (*N* = 619)	Male (*N* = 391)	Female (*N* = 228)	*P*-value
**Age**				
18−24	100 (16.2)	72 (18.4)	28 (12.3)	0.002
25−44	406 (65.6)	258 (66.0)	148 (64.9)	
45±	113 (18.26)	61 (15.6)	52 (22.81)	
**Marital status**				
Currently married	193 (31.2)	101 (25.8)	92 (40.4)	<0.001
Divorced/separated/widowed	112 (18.1)	51 (13.0)	61 (26.8)	
Never married	314 (50.7)	239 (61.1)	75 (32.9)	
**Occupation/employment status**				
Employed	362 (58.5)	242 (61.9)	120 (52.6)	0.024
Unemployed	257 (41.5)	149 (38.1)	108 (47.4)	
**Religious affiliation**				
Christian	402 (64.9)	220 (56.3)	182 (79.8)	<0.001
Other	217 (35.1)	171 (43.7)	46 (20.2)	
**Monthly household income**				
<10000	312 (54.6)	185 (50.7)	127 (61.7)	0.005
10,000−≤40,000	205 (35.9)	136 (37.3)	69 (33.5)	
>41,000	54 (9.5)	44 (12.1)	10 (4.9)	
**Family history of suicidality**				
Yes	150 (24.2)	87 (22.3)	63 (27.6)	0.132
No	469 (75.8)	304 (77.8)	165 (72.4)	
**Highest level of education**				
Education level 0−8 years	226 (36.5)	135 (34.5)	91 (39.9)	0.329
Formal education 9−13 years	258 (41.7)	171 (43.7)	87 (38.2)	
Formal education ≥ 14	135 (21.8)	85 (21.7)	50 (21.9)	
**Comorbid MDD**				
Yes	92 (15.5)	58 (15.6)	34 (15.3)	0.918
No	501 (84.5)	313 (84.4)	188 (84.7)	
**Comorbid bipolar disorder**				
Yes	391 (65.9)	231 (62.3)	160 (72.1)	0.015
No	202 (34.1)	140 (37.7)	62 (27.9)	
**Comorbid substance use**				
Yes	404 (65.3)	325 (83.1)	79 (34.7)	<0.001
No	215 (34.7)	66 (16.9)	149 (65.4)	
**Presence of chronic illness**				
Yes	335 (54.2)	186 (47.6)	149 (65.6)	<0.001
No	283 (45.8)	205 (52.4)	78 (34.4)	
**Number of negative life events**				
0−1	433 (70.0)	265 (67.8)	168 (73.7)	0.059
2−3	139 (22.5)	89 (22.7)	50 (21.9)	
4 or more	47 (7.6)	37 (9.5)	10 (4.4)	
**Negative symptoms**				
Yes	481 (81.1)	300 (80.8)	181 (81.5)	0.840
No	112 (18.8)	71 (19.1)	41 (18.4)	
**Symptoms of delusion**				
Yes	433 (70.0)	265 (67.7)	168 (73.6)	0.122
No	186 (30.0)	126 (32.2)	60 (26.3)	
**Auditory hallucinations**				
Yes	459 (77.4)	288 (77.6)	171 (77.0)	0.866
No	134 (22.6)	83 (22.3)	51 (22.9)	

### Frequency pattern and distribution of suicidality

Overall suicidal behavior, measured as any current SI or lifetime suicidal attempts, was reported by 203 (32.8%) participants with psychotic disorders, of whom 181 (29.2%) had a suicidal attempt in their history, 60 (9.7%) had SI in the past month, and 129 (20.9%) had both. When looking at SI and SB across patients with different types of symptoms, it was clear that SI and SB were highest among those with visual hallucinations (39%) and lowest in delusions of control/thoughts of broadcasting (32%) (Supplementary Table 2). SI and SB were more common in those with unemployed status (39.6% vs. 27.9%, *p* = 0.002), family history of suicide (46.0% vs. 28.5%, *p* < 0.001), comorbid bipolar disorder (36.0% vs. 26.1%, *p* = 0.005), negative symptoms (35.5 vs. 17.8%, *p* < 0.001), auditory hallucinations (34.86% vs. 23.1%, *p* = 0.011), and lifetime events (42.4% vs. 27.4%, *p* < 0.001) for 2–3 life events and (53.1% vs. 27.4%, *p* < 0.001) for 3–4 life events compared to those without these features (Supplementary Table 1).

In the univariate analysis, a family history of suicidality was associated with an increased risk for all three suicidality outcomes: odds ratio (OR) = 2.29 (95% CI:1.31–3.98) for SI, OR = 2.07 (95% CI:1.40–3.04) for suicidal attempts, and OR = 2.12 (95% CI:1.45–3.10) for SI and SB. Significant associations across all three outcomes were also found for a higher number of life events and the presence of negative symptoms. Comorbid substance use was associated with SI [OR = 2.04 (95%CI: 1.08–3.87)], but not with suicidal attempts and SI and SB (see Table 2).

**TABLE 2:** Univariate risk factors for suicidal ideation, suicidal attempts, and suicidal behavior (from univariate analyses), *N* = 619.

Characteristic	Association with suicidal ideation (SI): OR (95% CI)		Association with suicidal attempt: OR (95% CI)		Association with SI and SB: OR (95% CI)	
**Sex**	**OR (95% CI)**	***P*** **-value**	**OR (95% CI)**	***P*** **-value**	**OR (95% CI)**	***P*** **-value**
Female	1	0.153	1	0.625	1	0.623
Male	1.53 (0.85−2.76)		1.09 (0.76−1.57)		1.09 (0.76−1.54)	
**Age**						
18−24	1	0.005	1	0.104	1	0.026
25−44	0.38 (0.20−0.70)		0.76 (0.47−1.21)		0.65 (0.42−1.02)	
45+	0.32 (0.20−0.69)		0.61 (0.33−1.10)		0.52 (0.29−0.92)	
**Marital status**						
Currently married	1	0.367	1	0.336	1	0.367
Divorced/separated/widowed	0.66 (0.28−1.56)		1.50 (0.91−2.51)		1.46 (0.90−2.40)	
Never married	0.98 (0.54−1.77)		1.20 (0.83−1.88)		1.22 (0.83−1.81)	
**Occupation/employment status**						
Employed	1	0.095	1	0.022	1	0.002
Unemployed	1.56 (0.92−2.69)		1.50 (1.06−2.14)		1.70 (1.2−2.39)	
**Religion**						
Christian	1	0.783	1	0.993	1	0.834
Others	0.92 (0.53−1.60)		1.02 (0.71−1.46)		1.03 (0.73−1.50)	
**Monthly household income**						
<10,000	1	0.079	1	0.668	1	0.851
10,000−40,000	1.08 (0.58−1.99)		0.86 (0.55−1.22)		0.82 (0.56−1.20)	
>40,000	2.40 (1.08−5.30)		1.03 (0.55−1.92)		1.33 (0.74−2.41)	
**Family history of suicidality**						
No	1	0.003	1	<0.001	1	<0.001
Yes	2.29 (1.31−3.98)		2.07 (1.40−3.04)		2.12 (1.45−3.10)	
**Highest level of education**						
Level of education 1−8	1	0.974	1	0.525	1	0.482
Level of education 8−13	1.30 (0.71−2.37)		0.98 (0.66−1.45)		1.00 (0.68−1.46)	
Level of education ≥ 14	0.09 (0.06−0.15)		0.84 (0.52−1.35)		0.83 (0.52−1.31)	
**Comorbid MDD**						
No	1	0.835	1	0.398	1	0.262
Yes	0.90 (0.42−2.02)		0.80 (0.48−1.33)		0.75 (0.46−1.23)	
**Comorbid bipolar disorder**						
No	1	0.266	1	0.014	1	0.005
Yes	1.40 (0.76−2.63)		1.64 (1.11−2.43)		1.71 (1.17−2.50)	
**Comorbid substance use**						
No	1	0.028	1	0.068	1	0.059
Yes	2.04 (1.08−3.87)		1.41 (0.97−2.06)		1.42 (1.00−2.03)	
**Presence of chronic illness**						
No	1	0.138	1	0.985	1	0.306
Yes	1.52 (0.88−2.63)		1.00 (0.70−1.41)		1.19 (0.86−1.67)	
**Number of negative life events**						
0−1	1	0.014	1	<0.001	1	
2−3	2.03 (1.12−3.68)		1.95 (1.30−2.93)		1.94 (1.31−2.90)	<0.001
4 or more	2.12 (0.88−5.10)		3.30 (1.79−6.09)		3.00 (1.62−5.52)	
**Negative symptoms**						
No	1	0.028	1	0.001	1	<0.001
Yes	1.21 (1.02−1.44)		2.41 (1.40−4.13)		2.53 (1.51−4.26)	
**Delusions**						
No	1	0.760	1	0.398	1	0.192
Yes	1.05 (0.92−1.12)		1.17 (0.80−1.73)		1.28 (0.88−1.86)	
**Auditory hallucinations**						
No	1	0.072	1	0.014	1	0.011
Yes	1.13 (0.98−1.29)		1.79 (1.12−2.85)		1.77 (1.13−2.77)	

SI, suicidal ideation; SB, suicidal behavior; SI and SB, Aggregate of SI and/or suicidal attempts.

**FIGURE 2:**
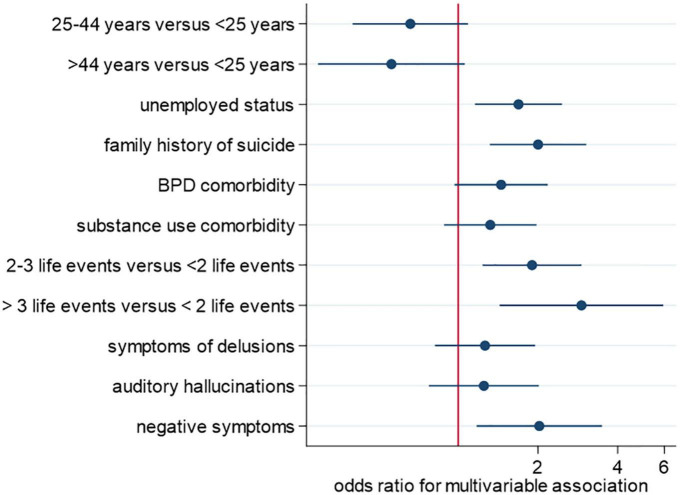
Multivariate factors for suicidal behavior.

In multivariate models, a family history of suicidality was significantly associated with an increased risk of suicidality across all the following three outcomes: SI [OR = 2.56 (95% CI: 1.34–4.88)], suicidal attempts [OR = 2.01 (95% CI: 1.31–3.06)], and SI and SB [OR = 2.00 (95% CI: 1.31–3.04)]. Compared to persons aged below 25 years, there were reduced odds for SI for persons aged ≥ 25 years [OR = 0.30 (95% CI: 0.14–0.62)] and persons aged ≥ 45 years [OR = 0.32 (95% CI: 0.12–0.89)]. Unemployment was associated with an increased risk for suicidal attempts [OR = 1.58 (95%CI: 1.08–2.33)] and SI and SB [OR = 1.68 (95% CI: 1.15–2.46)], but not SI. Similarly, the presence of negative symptoms increased the risk for suicidal attempts [OR = 2.02 (95%CI: 1.15−3.54)] and SI and SB [OR = 2.02 (95% CI: 1.21–2.46)]. Experiencing negative life events was associated with increased risk for both suicidal attempts and SI and SB. The strength of this association increased with an increasing number of life events experienced. For the SI and SB outcomes [OR = 1.89 (95% CI: 1.23–2.91)] for 2–3 negative life events and [OR = 2.91 (95% CI: 1.43–5.94)] for 4 or more negative life events (Figure 2 and Table 3). The AIC values for multivariate models were better than those for univariate models.

**TABLE 3:** Results from multivariate analysis of risk factors for suicidality, *N* = 619.

Characteristic	Association with suicidal ideation: OR (95% CI)		Association with suicidal attempts: OR (95% CI)		Association with SI and SB: OR (95% CI)	
**Sex**	**OR (95% CI)**	***P*** **-value**	**OR (95% CI)**	***P*** **-value**	**OR (95% CI)**	***P*** **-value**
Female	1	0.364	−	−	−	−
Male	1.42 (0.66−3.08)		−		−	
**Age**						
18−24	1	0.016	1	0.235	1	0.075
25−44	0.30 (0.14−0.62)		0.80 (0.48−1.34)		0.65 (0.39−1.08)	
45+	0.32 (0.12−0.89)		0.67 (0.34−1.29)		0.55 (0.29−1.05)	
**Occupation/employment status**						
Employed	1	0.976	1	0.019	1	0.007
Unemployed	1.01 (0.52−1.94)		1.58 (1.08−2.33)		1.68 (1.15−2.46)	
**Monthly household income**						
<10,000	1	0.055	−	−	−	−
10,000−40,000	1.09 (0.55−2.16)		−		−	
>40,000	2.81 (1.15−6.84)		−		−	
**Family history of suicidality**						
No	1	0.004	1	0.001	1	0.001
Yes	2.56 (1.34−4.88)		2.01 (1.31−3.06)		2.00 (1.31−3.04)	
**Comorbid bipolar disorder**						
No	−	−	1	0.121	1	0.072
Yes	−		1.39 (0.91−2.10)		1.46 (0.98−2.17)	
**Comorbid substance use**						
No	1	0.339	1	0.186	1	0.174
Yes	1.46 (0.66−3.20)		1.32 (0.87−1.99)		1.32 (0.88−1.97)	
**Presence of chronic illness**						
No	1	0.075	−	−	−	−
Yes	1.82 (0.94−3.52)		−		−	
**Number of negative life events**						
0−1	1	0.162	1	<0.001	1	<0.001
2−3	1.78 (0.88−3.57)		1.92 (1.24−2.96)		1.89 (1.23−2.91)	
4 or more	1.44 (0.50−4.18)		3.17 (1.57−6.38)		2.91 (1.43−5.94)	
**Negative symptoms**						
No	1	0.181	1	0.013	1	0.011
Yes	2.10 (0.70−6.23)		2.02 (1.15−3.54)		2.02 (1.17−3.49)	
**Symptoms of delusion**						
No	−	−	−	−	1	0.294
Yes	−		−		1.26 (0.81−1.95)	
**Auditory hallucinations**						
No	1	0.169			1.24 (0.77−2.01)	0.360
Yes	1.92 (0.75−4.82)					

Analyses included all variables with a *p*-value of ≤0.25 in univariate analyses. AIC model for suicidal behavior = 1.183; AIC model for SI = 0.594; AIC model for suicidal attempts = 1.135.

Unemployment had the highest population-attributable fraction for SI and SB outcomes [OR = 13.5% (95% CI: 5.6%–21.2%)] (Table 4).

**TABLE 4:** Population attributable risk percentage derived from multivariate model for suicidal behavior.

Risk factor	SI and SB: PAR% (95%CI)
Unemployed status	13.5% (5.6−21.2%)
Family history of suicide	3.5% (1.4−5.6%)
BPD comorbidity	4.9% (−1.5−10.1%)
Number of life events	4.8% (2.3−7.3%)
Negative symptoms	11.3% (4.0%−18.6%)
Age	Non-modifiable variable, no PAR computed

Findings from the multivariate linear regression model for the continuous risk score of suicide (the 16 items included) mirrored those for categorical suicidality outcomes above. The presence of a family history of suicide was associated with a higher risk for suicide [beta coefficient (β) = 7.04 (2.72; 11.36), *p* = 0.001], as was experiencing 2–3 negative life events [β = 9.20 (4.73; 13.66), *p* = 0.001]. The risk for suicide was also determined by age categories, being lower [β = –11.00 (–16.02; –5.97), *p* < 0.001] for ages 25–44 years, while ages 45 years and older were associated with a lower overall risk score for suicide [β = –19.24 (–19.56; –6.93) *p* < 0.001] as compared to the low age group of ≤24 years (Table 5).

**TABLE 5:** Univariate and multivariate linear regression analyses: Sociodemographic and clinical factors associated with an overall suicide risk score, *N* = 615.

Characteristic	Univariate association with suicide overall sum score beta (β) coefficient (95% CI)		Multivariable association with suicide overall sum score beta (β) coefficient (95% CI)	
**Sex**	**β coefficient (95% CI)**	***P*** **-value**	**β coefficient (95% CI)**	***P*** **-value**
Female	1	0.202	1	0.732
Male	2.60 (−1.39; 6.60)		−0.77 (−5.24; 3.68)	
**Age**				
18−24	1	<0.001	1	<0.001
25−44	−10.53 (−15.82; −5.25)		−11.00 (−16.02; −5.97)	
45+	−13.46 (−20.23; −7.20)		−13.24 (−19.56; −6.93)	
**Marital status**				
Currently married	1	0.837	−	−
Divorced/separated/widowed	2.37 (−3.32; 8.08)		−	−
Never married	0.64 (−3.75; 5.04)		−	−
**Occupation/employment status**				
Employed	1	0.372	−	−
Unemployed	1.78 (−2.13; 5.70)		−	−
**Religion**				
Christian	1	0.099	1	0.467
Others	−3.40 (−7.43; 0.63)		−1.46 (−5.41; 2.47)	
**Monthly household income**				
<10,000	1	0.582	−	−
10,000−40,000	−0.74 (−5.18; 3.69)		−	−
>40,000	3.67 (−3.60; 10.95)		−	−
**Family history of suicidality**				
No	1	<0.001	1	0.001
Yes	8.53 (4.08; 12.99)		7.04 (2.72; 11.36)	
**Highest level of education**				
Level of education 0−8	1	0.206	1	0.194
Level of education 8−13	0.02 (−4.36; 4.37)		−0.15 (−4.24; 4.21)	
Level of education ≥ 14	−3.73 (−8.95; 1.48)		−3.40 (−8.39; 1.58)	
**Comorbid MDD**				
No	1	0.671	−	−
Yes	−1.12 (−6.33; 4.07)		−	−
**Comorbid bipolar disorder**				
No	1	0.217	1	0.762
Yes	2.50 (−1.47; 6.47)		0.59 (−3.34; 4.53)	
**Comorbid substance use**				
No	1	0.004	1	0.190
Yes	5.84 (1.81; 9.87)		3.74 (−0.700.; 8.19)	
**Presence of chronic illness**				
No	1	0.068	1	0.111
Yes	3.60 (−0.27; 7.47)		3.04 (−0.69; 6.78)	
**Number of negative life events**				
0−1	1	<0.001	1	0.001
2−3	10.80 (6.21; 15.4)		9.20 (4.73; 13.66)	
4 or more	10.75 (3.52; 17.9)		5.75 (−1.71; 13.21)	
**Negative symptoms**				
No	1	0.008	1	0.100
Yes	6.45 (1.66; 11.24)		3.95 (−0.07; 8.67)	
**Symptoms of delusion**				
No	1	0.539	−	−
Yes	−1.31 (−5.53; 2.89)		−	
**Auditory hallucinations**				
No	1	0.015	1	0.225
Yes	5.57 (1.08; 10.06)		2.77 (−1.71; 7.25)	

## Discussion

This is a large study examining the risk of suicidality in psychosis in an African country. Suicidality is prevalent (32%) in outpatients with psychotic disorders recruited from hospitals on the Kenyan coast. Some risk factors were found in both suicidal attempts and ideations, for example, a family history of suicide, while others were only associated with one suicidality category, e.g., unemployment and younger age (<25 years) for SI and the presence of negative symptoms for suicidal attempts. Finally, an increasing number of negative life events increased the risk for both suicidal attempts and SI and SB. Public interventions targeted at reducing unemployment could have the most significant impact on decreasing SI and SB because they had higher PAR compared to other risk factors found in this study.

The prevalence of SI and SB was 32%, comparable to other study findings from high-income countries examining patients with schizophrenia and other psychotic disorders (30%) (29). The frequency of suicidal attempts in persons with schizophrenia has been found to range between 18% and 55% (10) significantly higher compared to the general population. The wide range across the literature can be explained by differences in populations (e.g., schizophrenia vs. other psychotic disorders) and methods (e.g., lifetime or recent suicidal behavior). A recent meta-analysis with 16,747 individuals diagnosed with schizophrenia globally found a pooled lifetime prevalence of suicidal attempts at 26% (30), only slightly lower than our suicide attempt prevalence of 29%. Studies from populations in sub–Saharan Africa on suicidal attempts in persons with schizophrenia are scarce. A study in Ethiopia found an SB prevalence of 30% (31), which is closely similar to our study (32%); however, in Nigeria, the prevalence of SB was higher than in our study at 43% (32). This higher prevalence was attributed to the very high levels of stigma for schizophrenia and psychotic disorders found in Nigeria (33). However, the variation can be explained by methodological differences; for example, our wider inclusion criteria of patients with psychotic disorders and mood disorders with psychotic disorders in contrast to the Nigerian study, whose inclusion was specific to patients with a diagnosis of schizophrenia disorder.

In our samples, there was some heterogeneity in the presence of different types of symptoms, with negative symptoms being the most common (81%), and delusions being the least common (70%). In our analysis, it was clear that the presence of negative symptoms showed the strongest link to SI and suicidal attempts, and in multivariate analyses, this was a consistently significant determinant of suicide. The presence of hallucinations and delusions were not further contributing to the suicide risk. The important role of negative symptoms is well described in the literature (34). Studies have reported poor social functioning and unstable social support as being more common in patients with negative symptoms, which in turn may increase the risk of SB (35). In contrast, some studies have found negative symptoms to be associated with a reduced risk of suicidality. It is hypothesized that depressive symptoms may modify this association (36).

Psychiatric comorbidity in other studies (37) is a consistent risk factor for suicide, particularly depression. However, we did not find major depressive disorder being associated with any of the suicidality outcomes, not even in univariate analyses. This inconsistency may be explained by the fact that our analysis included both current and past mood disorders. Current depressive disorders are more strongly associated with recent SB in patients with psychotic disorders (13). Further depressive episodes, as risk factors for suicide, have been reported to be most predictive of suicide in the early phases of psychotic disorders (1). Our study included patients with chronic psychotic disorders and was not limited to the first episode of patients with psychosis. Another explanation for this inconsistency may be related to the difficulty in recognizing major depressive disorder in patients with psychotic disorders, while the related psychological construct of demoralization, which is easier to identify, has the potential to influence SI and SB among these patients. In a recent systematic review, Darke et al. (38) highlighted the psychological construct of demoralization and its crucial link to suicidality. A subcomponent of demoralization is hopelessness, which may explain the higher rate of SI and SB at the onset of schizophrenia (39).

Comorbid alcohol and drug use disorders are strongly associated with suicide risk in persons with psychotic disorders (40). Previous studies have found that associations with suicide risk depend on types of substance use, e.g., alcohol for all mental health disorders and other nonalcoholic substances for bipolar disorders (41). Our study was not sufficiently powered to explore the influence of individual types of substances, given that all substance use regressed against the summated risk score outcomes, which have more power than binary outcomes. Participants in our study were drawn from the Kenyan coast, where substance abuse is rampant and illegal, so misreporting because of social desirability bias is possible (42).

Negative life events are antecedents for psychosocial distress and mental illnesses such as psychosis; thus, it is not surprising that these increased suicide risk in our study (43). The number of life events is positively associated with the risk of suicide in patients with psychotic disorders (44). This similar pattern is found with other mental disorders such as depression and bipolar mood disorder (45).

Male sex, a younger age, and a single marital status are demographic factors reported elsewhere to increase the risk of suicide in persons with psychotic disorders (16). In contrast, our study found no association between the male sex and elevated suicide risk. In many studies, suicide mortality is higher in men due to the lethality of means used (46); however, SI and SB are commonly higher in women than in men. However, this gender gap is less obvious in developing countries due to cultural factors such as power distance, individualism, uncertainty avoidance, and masculinity (47). These factors may play a role in the equal distribution of suicide risk seen between the two sexes in our study. Unlike in other suicide risk studies, marital status was not associated with suicide risk in our study. Married status is considered a proxy measure for a social support network, of which the latter is a better metric (29). In African culture, social interaction and group-living is common, and may thus have extended benefits to unmarried patients with psychotic disorders. However, in our study, young age was also a consistent risk factor for suicidal ideation and overall suicide risk. This is similar to other study findings (48, 49). The change in status from a healthy life and the recognition of the burden of a chronic debilitating diagnosis has been postulated as one reason for this. The reduction in suicide risk with increasing age has been thought to be related to functional recovery, learned adaptations to living with the symptoms of psychosis, stabilization, and improvement of symptoms seen in some older patients with psychotic disorders (50).

Being unemployed increased suicide risk in our study cohort and is likely a measure of poor economic status. Other studies have argued that mental illnesses, specifically psychotic disorders, may be a potential confounder for unemployment (51). This study still finds a strong link between suicide risk with unemployment among patients with psychotic disorders. The increased suicide risk linked with unemployment may be through the vicious cycle of social causation, which is psychological stress from poverty leading to an inability to access mental health services and selection brought on by stigma related to mental illness diagnosis, leading back to reduced access to employment (52). In the face of economic hardships such as those experienced in LMIC, interpersonal trust is significantly protective of SI and SB (53). However, even though interpersonal trust and networking are more developed in LMICs compared to HICs, patients with schizophrenia in sub-Saharan Africa are less likely to benefit from this due to the existing higher levels of stigma and isolation of these patients. However, the benefit of interpersonal trust in reducing suicides in LMICs may be complicated by the economic crises related to the COVID-19 pandemic (54). Since unemployed status had the highest PAR, measures to decrease poverty may reduce the incidence of suicide in persons with psychotic disorders.

Family history of suicide was consistently significant across all suicidality outcomes. It may point to two possibilities, namely, shared environmental risk factors or underlying genetic susceptibility. The environmental risk factors may be through the generation transmission of adverse environments, e.g., poverty (55), or learned poor coping mechanisms to psychological stressors, e.g., bereavement or direct imitation of destructive behavior (56). For genetic susceptibility, a recent meta-analysis revealed the following two loci associated with suicidal attempts: the major histocompatibility complex and an intergenic locus on chromosome 7 (57).

### Addressing suicide in persons with psychosis

To prevent suicide in persons with psychotic disorders, clinicians should be able to identify both population and individual suicide risk factors in their patients (16). Our study found that being unemployed, having a family history of suicidality, experiencing several negative life events, and the presence of negative symptoms of psychosis are important risk factors that should be considered in the suicide assessment of patients with psychotic disorders in sub-Saharan Africa. Explicit assessment of suicidal ideation in patients with schizophrenia, though crucial for proper management of these patients, is unfortunately often overlooked in clinical practice. In this regard, brief screening using specific scales such as the Scale for Suicidal Ideation (SIS) can be integrated into the clinical assessment of patients with schizophrenia (58, 59). Mental health systems strengthening that incorporates training clinicians on the proper management of psychotic disorders including identification and treatment of comorbid conditions such as mood and substance use disorders can reduce suicidality in persons with psychotic disorders. Targeting modifiable risk factors, for example, economic empowerment and stigma reduction, at the workplace, can be useful in addressing existing high rates of unemployment in this especially vulnerable group (60). Even beyond the workplace, stigma reduction approaches and strategies, such as mental health literacy campaigns and policy and legislative changes, can be impactful in improving access to needed care (61). Recently published exploratory findings in a similar study population confirmed the impact of suicide and mental illness-related stigma on treatment-seeking delays and adherence (62). Severe mental illnesses, such as psychotic disorders, can be isolating, thereby worsening disability and limiting social and occupational functioning if support and treatment are insufficient.

### Strengths and limitations

Our study has several strengths. We assessed a range of demographic and clinical factors associated with suicidality in a large sample of 619 individuals diagnosed with psychotic disorders. We use the current DSM-5 diagnostic criteria for psychotic disorders for study eligibility. Our assessments of comorbidities, such as chronic illness, bipolar mood disorder, major depressive mood disorders, and substance use disorders, are made using standardized tools. In addition, we used a validated 16-item instrument to assess suicidality. Another strength is that most of the potential participants approached consented to be in the study; the response rate was high. In addition, we attempted to undertake a random selection of participants from the parent study based on dates of recruitment in the primary study. Finally, we were able to use both categorical and continuous measures to assess the risk of suicidality in this patient cohort group. We acknowledge a few limitations. First, this was a cross-sectional study, which does not allow for the direction and causality of associations. Even though we have a large sample size of patients with psychotic disorders, our study was not powerful enough to analyze individual subgroups of psychotic disorders, e.g., schizophrenia or schizoaffective disorder. We did not include inpatients in our study sample; as these are patients with severe illness, suicidality prevalence may have been different from our current findings. In addition, our results may be influenced by response or selection bias. Finally, the biological basis of suicide in psychosis was not investigated in this study, and this warrants future research.

## Conclusion

Suicidal ideation and SB are common among outpatients with psychotic disorders in the coastal region of Kenya. This underscores the need for a thorough suicidality assessment in patients with psychotic disorders and risk factors associated with SB in persons with psychotic disorders. Unemployment, younger age, a family history of suicidality, and a higher number of negative life events were most prominently associated with an elevated risk for suicidality in this patient cohort. This suggests that suicide prevention interventions focused on economic empowerment and improved access to mental healthcare are warranted in this patient cohort. Future studies can subset patients with psychotic disorders to better understand suicide risk in the first episode of patients with psychosis compared to patients with chronic psychosis.

## Data availability statement

The raw data supporting the conclusions of this article will be made available by the authors, without undue reservation.

## Ethics statement

The studies involving human participants were reviewed and approved by the Kenya Medical Research Institute Scientific and Ethics Review Unit (SERU:3575 and SERU:3916) for the parent study and the sub study, respectively. The patients/participants provided their written informed consent to participate in this study.

## Author contributions

LO, SK, JT, BP, CS, and CN conceptualized, designed the study, and planned the statistical analysis. LO, MN, and SK analyzed the data. LO and SK wrote the first draft of the manuscript. All authors contributed to the interpretation and subsequent edits of the manuscript.

## References

[1] Bertelsen M Jeppesen P Petersen L Thorup A Øhlenschlæger J le Quach P Suicidal behaviour and mortality in first-episode psychosis: the OPUS trial. *Br J Psychiatry Suppl.* (2007) 51:s140–6. 10.1192/BJP.191.51.S140 18055932

[2] Siris S. Suicide and schizophrenia. *J Psychopharmacol.* (2001) 15:127–35. 10.1177/026988110101500209 11448086

[3] Bushe C Taylor M Haukka J. Mortality in schizophrenia: a measurable clinical endpoint. *J Psychopharmacol.* (2010) 24:17. 10.1177/1359786810382468 20923917 PMC2951589

[4] Skodlar B Tomori M Parnas J. Subjective experience and suicidal ideation in schizophrenia. *Compr Psychiatry.* (2008) 49:482–8. 10.1016/J.COMPPSYCH.2008.02.008 18702934

[5] Ventriglio A Gentile A Bonfitto I Stella E Mari M Steardo L Suicide in the early stage of schizophrenia. *Front Psychiatry.* (2016) 7:116. 10.3389/FPSYT.2016.00116 27445872 PMC4921745

[6] Khasakhala L Ndetei D Mathai M. Suicidal behaviour among youths associated with psychopathology in both parents and youths attending outpatient psychiatric clinic in Kenya. *Ann Gen Psychiatry.* (2013) 12:13. 10.1186/1744-859X-12-13 23622559 PMC3644274

[7] Sankoh O Sevalie S Weston M. Mental health in Africa. *Lancet Glob Health.* (2018) 6:e954–5. 10.1016/S2214-109X(18)30303-6 30103990

[8] Nordentoft M Laursen T Agerbo E Qin P Høyer E Mortensen P. Change in suicide rates for patients with schizophrenia in Denmark, 1981-97: nested case-control study. *BMJ.* (2004) 329:261. 10.1136/BMJ.38133.622488.63 15213108 PMC498022

[9] Drake R Whitaker A Gates C Cotton P. Suicide among schizophrenics: a review. *Compr Psychiatry.* (1985) 26:90–100. 10.1016/0010-440X(85)90053-7 3881217

[10] Sher L Kahn R. Suicide in schizophrenia: an educational overview. *Medicina.* (2019) 55:361. 10.3390/MEDICINA55070361 31295938 PMC6681260

[11] Thonney J Conus P Golay P. [Sexual and physical abuse during childhood; what is the impact on outcome in first episode psychosis patients?]. *Encephale.* (2021) 47:215–20. 10.1016/J.ENCEP.2020.06.010 33902898

[12] Kjelby E Sinkeviciute I Gjestad R Kroken R Løberg E Jørgensen H Suicidality in schizophrenia spectrum disorders: the relationship to hallucinations and persecutory delusions. *Eur Psychiatry.* (2015) 30:830–6. 10.1016/j.eurpsy.2015.07.003 26443050

[13] Large M Smith G Sharma S Nielssen O Singh S. Systematic review and meta-analysis of the clinical factors associated with the suicide of psychiatric in-patients. *Acta Psychiatr Scand.* (2011) 124:18–9. 10.1111/J.1600-0447.2010.01672.X 21261599

[14] Andriopoulos I Ellul J Skokou M Beratis S. Suicidality in the “prodromal” phase of schizophrenia. *Compr Psychiatry.* (2011) 52:479–85. 10.1016/J.COMPPSYCH.2010.10.011 21185016

[15] Kavanagh D McGrath J Saunders J Dore G Clark D. Substance misuse in patients with schizophrenia: epidemiology and management. *Drugs.* (2002) 62:743–55. 10.2165/00003495-200262050-00003 11929329

[16] Berardelli I Rogante E Sarubbi S Erbuto D Lester D Pompili M. The importance of suicide risk formulation in schizophrenia. *Front Psychiatry.* (2021) 12:2137. 10.3389/FPSYT.2021.779684/BIBTEX PMC8716825 34975579

[17] Ongeri L McCulloch C Neylan T Bukusi E Macfarlane S Othieno C Suicidality and associated risk factors in outpatients attending a general medical facility in rural Kenya. *J Affect Disord.* (2018) 225:413–21. 10.1016/j.jad.2017.08.059 28850856 PMC5663198

[18] Ongeri L Kirui F Muniu E Manduku V Kirumbi L Atwoli L Khat use and psychotic symptoms in a rural Khat growing population in Kenya: a household survey. *BMC Psychiatry.* (2019) 19:137. 10.1186/s12888-019-2118-3 31064338 PMC6505064

[19] Stevenson A Akena D Stroud R Atwoli L Campbell M Chibnik L Neuropsychiatric genetics of African populations-psychosis (NeuroGAP-Psychosis): a case-control study protocol and GWAS in Ethiopia, Kenya, South Africa and Uganda. *BMJ Open.* (2019) 9:e025469. 10.1136/bmjopen-2018-025469 30782936 PMC6377543

[20] Cardno A Owen M. Genetic relationships between schizophrenia, bipolar disorder, and schizoaffective disorder. *Schizophr Bull.* (2014) 40:504–15. 10.1093/SCHBUL/SBU016 24567502 PMC3984527

[21] Sheehan DV Lecrubier Y Sheehan K Amorim P Janavs J Weiller E The mini-international neuropsychiatric interview (M.I.N.I.): the development and validation of a structured diagnostic psychiatric interview for DSM-IV and ICD-10. *J Clin Psychiatry.* (1998) 59(Suppl. 2):22–33. 9881538

[22] Sheehan D Lecrubier Y Harnett Sheehan K Janavs J Weiller E Keskiner A The validity of the mini international neuropsychiatric interview (MINI) according to the SCID-P and its reliability. *Euro Psychiatry.* (1997) 12:232–41. 10.1016/S0924-9338(97)83297-X

[23] Roaldset J Linaker O Bjørkly S. Predictive validity of the MINI suicidal scale for self-harm in acute psychiatry: a prospective study of the first year after discharge. *Arch Suicide Res.* (2012) 16:287–302. 10.1080/13811118.2013.722052 23137219

[24] Gray M Litz B Hsu J Lombardo T. Psychometric properties of the life events checklist. *Assessment.* (2004) 11:330–41. 10.1177/1073191104269954 15486169

[25] Wittchen H Nelson C. The composite international diagnostic interview: an instrument for measuring mental health outcome? In: Thornicroft G Tansella M editors. *Mental Health Outcome Measures.* (Berlin: Springer) (1996). p. 179–87. 10.1007/978-3-642-80202-7_13

[26] Who Assist Working Group. The alcohol, smoking and substance involvement screening test (ASSIST): development, reliability and feasibility. *Addiction.* (2002) 97:1183–94. 12199834 10.1046/j.1360-0443.2002.00185.x

[27] StataCorp. *Stata Statistical Software: Release 17.* College Station, TX: StataCorp (2021).

[28] Ferré J. Regression diagnostics. *Compr Chemometr.* (2009) 3:33–89. 10.1016/B978-044452701-1.00076-4

[29] Radomsky E Haas G Mann J Sweeney J. Suicidal behavior in patients with schizophrenia and other psychotic disorders. *Am J Psychiatry.* (1999) 156:1590–5. 10.1176/AJP.156.10.1590/ASSET/IMAGES/LARGE/AR20T2.JPEG 10518171

[30] Lu L Dong M Zhang L Zhu X Ungvari G Ng C Prevalence of suicide attempts in individuals with schizophrenia: a meta-analysis of observational studies. *Epidemiol Psychiatr Sci.* (2020) 29:e39. 10.1017/S2045796019000313 31172899 PMC8061230

[31] Ayalew M Defar S Reta Y. Suicide behavior and its predictors in patients with schizophrenia in ethiopia. *Schizophr Res Treat.* (2021) 2021:6662765. 10.1155/2021/6662765 33868728 PMC8032509

[32] Ogunnubi OP Aina FO Busari CO Fatiregun O Fadipe B Adegbohun AA From ideation to attempt: a study of suicidality and its correlates amongst patients with schizophrenia in a resource-poor country. *S Afr J Psychiat.* (2022) 28:a1547. 10.4102/sajpsychiatry.v28i0.1547 35169504 PMC8831998

[33] Fadipe B Adebowale T Ogunwale A Fadipe Y Ojeyinka A Olagunju A. *Internalized stigma in schizophrenia: a cross-sectional study of prevalence and predictors.* Hamilton, ON: McMaster University (2018). p. 1–12. 10.1080/17542863.2018.1450431

[34] Tarrier N Barrowclough C Andrews B Gregg L. Risk of non-fatal suicide ideation and behaviour in recent onset schizophrenia–the influence of clinical, social, self-esteem and demographic factors. *Soc Psychiatry Psychiatr Epidemiol.* (2004) 39:927–37. 10.1007/S00127-004-0828-3 15549247

[35] Jahn D Bennett M Park S Gur R Horan W Kring A The interactive effects of negative symptoms and social role functioning on suicide ideation in individuals with schizophrenia. *Schizophr Res.* (2016) 170:271. 10.1016/J.SCHRES.2015.12.011 26746862 PMC4762008

[36] Grover L Jones R Bass N McQuillin A. The differential associations of positive and negative symptoms with suicidality. *Schizophr Res.* (2022) 248:42–9. 10.1016/J.SCHRES.2022.07.016 35933743

[37] Carlborg A Winnerbäck K Jönsson E Jokinen J Nordstrm P. Suicide in schizophrenia. *Expert Rev Neurother.* (2010) 10:1153–64. 10.1586/ERN.10.82 20586695

[38] Costanza A Vasileios C Ambrosetti J Shah S Amerio A Aguglia A Demoralization in suicide: a systematic review. *J Psychosom Res.* (2022) 157:110788. 10.1016/J.JPSYCHORES.2022.110788 35334350

[39] Clarke D Kissane D. Demoralization: its phenomenology and importance. *Aust N Z J Psychiatry.* (2016) 36:733–42. 10.1046/J.1440-1614.2002.01086.X 12406115

[40] Gut-Fayand A Dervaux A Olié J Lôo H Poirier M Krebs M. Substance abuse and suicidality in schizophrenia: a common risk factor linked to impulsivity. *Psychiatry Res.* (2001) 102:65–72. 10.1016/S0165-1781(01)00250-5 11368841

[41] Østergaard M Nordentoft M Hjorthøj C. Associations between substance use disorders and suicide or suicide attempts in people with mental illness: a Danish nation-wide, prospective, register-based study of patients diagnosed with schizophrenia, bipolar disorder, unipolar depression or personality disorder. *Addiction.* (2017) 112:1250–9. 10.1111/ADD.13788 28192643

[42] Sloan J Bodapati M Tucker T. Respondent misreporting of drug use in self-reports: social desirability and other correlates. *J Drug Issues.* (2016) 34:269–92. 10.1177/002204260403400202

[43] Beards S Gayer-Anderson C Borges S Dewey M Fisher H Morgan C. Life events and psychosis: a review and meta-analysis. *Schizophr Bull.* (2013) 39:740–7. 10.1093/SCHBUL/SBT065 23671196 PMC3686461

[44] Fennig S Horesh N Aloni D Apter A Weizman A Fennig S. Life events and suicidality in adolescents with schizophrenia. *Eur Child Adolesc Psychiatry.* (2005) 14:454–60. 10.1007/S00787-005-0498-Z 16341502

[45] Horesh N Nachshoni T Wolmer L Toren P. A comparison of life events in suicidal and nonsuicidal adolescents and young adults with major depression and borderline personality disorder. *Compr Psychiatry.* (2009) 50:496–502. 10.1016/J.COMPPSYCH.2009.01.006 19840586

[46] Mergl R Koburger N Heinrichs K Székely A Tóth M Coyne J What are reasons for the large gender differences in the lethality of suicidal acts? An epidemiological analysis in four european countries. *PLoS One.* (2015) 10:e0129062. 10.1371/journal.pone.0129062 26147965 PMC4492725

[47] Webster Rudmin F Ferrada-Noli M Skolbekken J. Questions of culture, age and gender in the epidemiology of suicide. *Scand J Psychol.* (2003) 44:373–81. 10.1111/1467-9450.00357 12887559

[48] Cassidy R Yang F Kapczinski F Passos I. Risk Factors for suicidality in patients with schizophrenia: a systematic review, meta-analysis, and meta-regression of 96 studies. *Schizophr Bull.* (2018) 44:787. 10.1093/SCHBUL/SBX131 29036388 PMC6007264

[49] Olfson M Stroup T Huang C Wall M Crystal S Gerhard T. Suicide Risk in medicare patients with schizophrenia across the life Span. *JAMA Psychiatry.* (2021) 78:876–85. 10.1001/JAMAPSYCHIATRY.2021.0841 34037667 PMC8156163

[50] Shepherd S Depp C Harris G Halpain M Palinkas L Jeste D. Perspectives on schizophrenia over the lifespan: a qualitative study. *Schizophr Bull.* (2012) 38:295–303. 20603443 10.1093/schbul/sbq075 PMC3283145

[51] Blakely T Collings S Atkinson J. Unemployment and suicide. Evidence for a causal association? *J Epidemiol Community Health.* (2003) 57:594–600. 12883065 10.1136/jech.57.8.594 PMC1732539

[52] Lund C Breen A Flisher A Kakuma R Corrigall J Joska J Poverty and common mental disorders in low and middle income countries: a systematic review. *Soc Sci Med.* (2010) 71:517–28. 10.1016/j.socscimed.2010.04.027 20621748 PMC4991761

[53] Economou M Madianos M Peppou L Theleritis C Patelakis A Stefanis C. Suicidal ideation and reported suicide attempts in Greece during the economic crisis. *World Psychiatry.* (2013) 12:53. 10.1002/WPS.20016 23471802 PMC3619166

[54] Costanza A Amerio A Aguglia A Serafini G Amore M Macchiarulo E From “the interpersonal theory of suicide” to “the interpersonal trust”: an unexpected and effective resource to mitigate economic crisis-related suicide risk in times of covid-19? *Acta Bio Med.* (2021) 92:2021417. 10.23750/ABM.V92IS6.12249 34739460 PMC8851025

[55] Brent D Melhem N. Familial transmission of suicidal behavior. *Psychiatr Clin North Am.* (2008) 31:157–77. 10.1016/J.PSC.2008.02.001 18439442 PMC2440417

[56] Burke A Galfalvy H Everett B Currier D Zelazny J Oquendo M Effect of exposure to suicidal behavior on suicide attempt in a high-risk sample of offspring of depressed parents. *J Am Acad Child Adolesc Psychiatry.* (2010) 49:114–21. 10.1097/00004583-201002000-00005 20215933 PMC2915586

[57] Mullins N Kang J Campos A Coleman J Edwards A Galfalvy H Dissecting the shared genetic architecture of suicide attempt, psychiatric disorders, and known risk factors. *Biol Psychiatry.* (2022) 91:313–27. 10.1016/J.BIOPSYCH.2021.05.029/ATTACHMENT/AB08ECA9-28FE-456E-BAE6-5D394B7AA675/MMC3.XLSX 34861974 PMC8851871

[58] Beck A Brown G Steer R. Psychometric characteristics of the scale for suicide ideation with psychiatric outpatients. *Behav Res Ther.* (1997) 35:1039–46. 9431735 10.1016/s0005-7967(97)00073-9

[59] Baertschi M Costanza A Canuto A Weber K. The dimensionality of suicidal ideation and its clinical implications. *Int J Methods Psychiatr Res.* (2019) 28:e1755. 10.1002/MPR.1755 30426604 PMC6877148

[60] Holm M Taipale H Tanskanen A Tiihonen J Mitterdorfer-Rutz E. Employment among people with schizophrenia or bipolar disorder: a population-based study using nationwide registers. *Acta Psychiatr Scand.* (2021) 143:61–71. 10.1111/ACPS.13254 33155273 PMC7839734

[61] World Health Organization. *WHO | Preventing Suicide: A Global Imperative.* Geneva: WHO Press (2014).

[62] Javed A Lee C Zakaria H Buenaventura R Cetkovich-Bakmas M Duailibi K Reducing the stigma of mental health disorders with a focus on low- and middle-income countries. *Asian J Psychiatr.* (2021) 58:102601. 10.1016/J.AJP.2021.102601 33611083

