# Supplementary material for: Suicidal attempts and ideations in Kenyan adults with psychotic disorders: An observational study of frequency and associated risk factors
Source: Front Psychiatry. 2023 Jan 19;13:1085201. doi: 10.3389/fpsyt.2022.1085201 (PMC9892760; doi:10.3389/fpsyt.2022.1085201)
Supplement: Supplementary file 1 [file Table_1.DOCX]

**Supplementary Table 1. Demographics Characteristics of outpatients with DSM 5 psychotic disorders by current (past 1 month) suicidal ideation, lifetime attempt and suicidal behavior (ideation and/or lifetime attempt) status**

| C**haracteristics** | **Suicidal Thoughts and Behavior** | | | | | | | | |
| --- | --- | --- | --- | --- | --- | --- | --- | --- | --- |
|  | **Suicidal ideation (SI):**  **N= 60(9.7%)** | **No suicidal ideation:**  **N=559(90.3%)** | **P-value** | **Lifetime Suicidal attempt: N=181(29.2)** | **No lifetime suicidal attempt: N=438(70.8)** | **P-value** | **SI & SB : N= 203(32.8)** | **SI & SB: N=416(67.2)** | **P value** |
| **Sex** |  |  |  |  |  |  |  |  |  |
| Male | 43(11.00) | 348(89.00) | 0.151 | 117(29.92) | 274(70.08) | 0.625 | 131(33.50) | 260(66.50) | 0.623 |
| Female | 17(7.46) | 211(92.54) |  | 64(28.07) | 164(71.93) |  | 72(31.58) | 156(68.42) |  |
| **Age** |  |  |  |  |  |  |  |  |  |
| 18-24 | 19(19.00) | 81(81.00) | 0.003 | 35(35.00) | 65(65.00) | 0.260 | 42(42.00) | 58(58.00) | 0.066 |
| 25-44 | 33(8.13) | 373(91.87) |  | 118(29.06) | 288(70.94) |  | 130(32.02) | 276(67.98) |  |
| 45+ | 8(7.08) | 105(92.92) |  | 28(24.78) | 85(75.22) |  | 31(27.43) | 82(72.57) |  |
| **Marital Status** |  |  |  |  |  |  |  |  |  |
| Currently Married | 20(10.36) | 173(89.64) | 0.601 | 49(25.39) | 144(74.61) | 0.266 | 56(29.02) | 137(70.98) | 0.296 |
| Separated/divorced/widowed | 8(7.14) | 104(92.86) |  | 38(33.93) | 74(66.079) |  | 42(37.50) | 70(62.50) |  |
| Never married | 32(10.36) | 282(89.81) |  | 94(29.943) | 22(70.06) |  | 105(33.44) | 209(66.56) |  |
| **Occupation/Employment Status** |  |  |  |  |  |  |  |  |  |
| Employed | 29(8.01) | 333(91.99) | 0.093 | 93(25.69) | 269(74.31) | 0.021 | 101(27.90) | 261(72.10) | 0.002 |
| Unemployed | 31(12.06) | 226(87.94) |  | 88(34.24) | 169(65.76) |  | 102(39.69) | 155(60.31) |  |
| **Religion** |  |  |  |  |  |  |  |  |  |
| Christian | 38(9.45) | 364(90.55) | 0.783 | 118(29.35) | 284(70.65) | 0.933 | 133(33.08) | 269(66.92) | 0.834 |
| Others | 22(10.14) | 195(89.86) |  | 63(29.03) | 154(70.97) |  | 70(32.26) | 147(67.74) |  |
| **Monthly Household Income** |  |  |  |  |  |  |  |  |  |
| <10,000 | 27(8.65) | 285(91.35) | 0.075 | 96(30.77) | 216(69.23) | 0.593 | 106(33.97) | 206(66.03) | 0.277 |
| 10,000-≤40,000 | 19(9.27) | 186(90.73) |  | 55(26.83) | 150(73.17) |  | 61(29.76) | 144(70.24) |  |
| >40,000 | 10(18.52) | 44(81.48) |  | 17(31.48) | 37(68.52) |  | 22(40.74) | 32(59.26) |  |
|  |  |  |  |  |  |  |  |  |  |
| **Family history of suicidality** |  |  |  |  |  |  |  |  |  |
| Yes | 24(16.00) | 126(84.00) | 0.003 | 62(41.33) | 88(58.67) | <0.001 | 69(46.00) | 81(54.00) | <0.001 |
| No | 36(7.68) | 433(92.32) |  | 119(25.37) | 350(74.63) |  | 134(28.57) | 335(71.43) |  |
|  |  |  |  |  |  |  |  |  |  |
| **Highest level of Education** |  |  |  |  |  |  |  |  |  |
| Level of education 0-8 | 20(8.85) | 206(91.15) | 0.533 | 68(30.09) | 158(69.91) | 0.757 | 76(33.63) | 150(66.37) | 0.675 |
| Level of education 8-13 | 29(11.24) | 229(88.76) |  | 77(29.84) | 181(70.16) |  | 87(33.72) | 171(66.28) |  |
| Level of education ≥14 | 11(8.15) | 124(91.85) |  | 36(26.67) | 99(73.33) |  | 40(29.63) | 95(70.37) |  |
| **Comorbid MDD** |  |  |  |  |  |  |  |  |  |
| Yes | 8(8.70) | 454(90.62) | 0.835 | 23(25.00) | 69(75.00) | 0.397 | 25(27.17) | 67(72.83) | 0.261 |
| No | 47(9.38) | 84(91.30) |  | 147(29.34) | 354(70.66) |  | 166(33.13) | 335(66.87) |  |
| **Comorbid Bipolar Disorder** |  |  |  |  |  |  |  |  |  |
| Yes | 40(10.23) | 351(89.77) | 0.265 | 125(31.97) | 266(68.03) | 0.013 | 141(36.06) | 250(63.94) | 0.005 |
| No | 15(7.43) | 187(92.57) |  | 45(22.28) | 157(77.72) |  | 50(24.75) | 152(75.25) |  |
| **Comorbid Substance use** |  |  |  |  |  |  |  |  |  |
| Yes | 47(11.63) | 357(88.37)) | 0.025 | 128(31.68) | 276(68.32) | 0.067 | 60(27.91) | 155(72.09) | 0.059 |
| No | 13(6.05) | 202(93.95) |  | 53(24.65) | 162(75.35) |  | 143(35.40) | 261(64.60) |  |
| **Presence of chronic illness** |  |  |  |  |  |  |  |  |  |
| Yes | 38(11.34) | 297(88.66) | 0.135 | 98(29.25) | 237(70.75) | 0.984 | 116(34.63) | 219(65.37) | 0.306 |
| No | 22(7.77) | 261(92.23) |  | 83(29.33) | 200(70.67) |  | 87(30.74) | 196(69.26) |  |
| **Number of Life events** |  |  |  |  |  |  |  |  |  |
| 0-1 | 33(7.62) | 400(92.38) | 0.029 | 104(24.02) | 329(75.98) | <0.001 | 119(27.48) | 314(75.52) | <0.001 |
| 2-3 | 20(14.39) | 119(85.61) |  | 53(38.138) | 86(61.87) |  | 59(42.45) | 80(57.55) |  |
| 4 or more | 7(14.89) | 40(85.11) |  | 24(51.06) | 23(48.94) |  | 25(53.19) | 22(46.81) |  |
| **Negative symptoms** |  |  |  |  |  |  |  |  |  |
| Yes | 51(10.60) | 430(89.40) | 0.021 | 152(31.60) | 329(68.40) | 0.001 | 171(35.55) | 310(64.45) | <0.001 |
| No | 4(3.57) | 108(96.43) |  | 18(16.07) | 94(83.93) |  | 20(17.86) | 92(82.14) |  |
| **Symptoms of Delusion** |  |  |  |  |  |  |  |  |  |
| Yes | 43(9.93) | 390(90.07) | 0.760 | 131(30.25) | 302(69.75) | 0.398 | 149(34.41) | 284(65.59) | 0.191 |
| No | 17(9.14) | 169(90.86) |  | 50(26.88) | 136(73.12) |  | 54(29.03) | 132(70.97) |  |
| **Auditory Hallucinations** |  |  |  |  |  |  |  |  |  |
| Yes | 48(10.46)) | 411(89.54) | 0.066 | 143(31.15) | 316(68.85) | 0.013 | 160(34.86) | 299(65.14) | 0.011 |
| No | 7(5.22 | 127(94.78) |  | 27(15.88) | 107(79.85) |  | 31(23.13) | 103(76.87) |  |

**Supplementary Table 2: Distribution of suicidality across selected persons with specific psychotic symptoms, and in the overall group**

| **SI & SB and suicide risk** | **Overall:**  **N=619** | **Paranoid Delusions:**  **N=390 (65%)** | **Delusions of control/ Thought of broadcasting:**  **N=450 (75.8%)** | **Auditory hallucinations:**  **N=459 (77.4%)** | **Visual hallucinations:**  **N=288 (48.5%)** | **Negative symptoms:**  **481 (81.11%)** |
| --- | --- | --- | --- | --- | --- | --- |
| **Current suicidal ideation(SI) (% yes, CI)** | 9.7% (7.4-12.3%) | 11.0%  (8.0-14.5%) | 9.7% (7.2-12.9%) | 10.4% (7.8-13.6%) | 10.4%  (7.1-14.5) | 10.6%  (7.9-13.7%) |
| **Lifetime suicidal attempt (% yes, CI)** | 29.2% (25.6-32.9%) | 31.5%  (26.9-36.4%) | 30.8% (26.6-35.3%) | 31.2% (.9-35.6%) | 35.7%  (30.2-41.5%) | 31.6%  (27.4-35.9%) |
| **SI & SB (ideation or attempt) (% yes, CI)** | 32.7% (29.1-36.6%) | 36.6%  (31.8-41.6%) | 34.2% (29.8-38.8%) | 34.8% (.4-39.4%) | 39.2%  (33.5-45.1%) | 35.5%  (31.2-40.0%) |
| **Moderate suicidal risk (% scoring 9-16, CI))** | 2.9% (1.7-4.5%) | 3.3%  (1.7-5.6%) | 3.1% (1.7-5.16%) | 2.3% (1.2-4.2%) | 2.4%  (0.9-4.9%) | 3.1% (  1.7-5.0%) |
| **High Suicidal Risk (% scoring ≥17, CI)** | 14.3% (11.7-17.3%) | 15.8% (12.4-19.9%) | 14.6(11.5-18.2%) | 15.0% (11.8-18.6%) | 15.6%  (11.6-20.3%) | 15.8%  (12.6-19.3%) |

**Supplementary Table 3: Distribution of psychotic disorders and comorbidities**

| **Diagnostic Category** | **N=619 (%)** |
| --- | --- |
| Psychotic disorders only | 28 (4.5) |
| Psychotic disorders with comorbid substance use | 109 (17.6) |
| Psychotic disorders with comorbid bipolar disorder | 154 (24.8) |
| Psychotic disorders with comorbid major depressive disorder | 33 (5.3) |
| Psychotic disorders with comorbid substance use and major depressive disorder | 58 (9.3) |
| Psychotic disorder with comorbid substance use and bipolar disorder | 237 (38.2) |

**Supplementary Table 4. Socio-demographic and clinical characteristics of study population by suicide risk level.**

|  | **Suicide risk level** | | |
| --- | --- | --- | --- |
| **Characteristic** | **Low risk (≤8) N=512** | **Moderate to high risk ≥9**  **N=107** | **P value** |
| **Sex** |  |  |  |
| Male | 318(62.11) | 27(30.34) | 0.173 |
| Female | 194(37.89) | 62(69.66) |  |
| **Age** |  |  |  |
| 18-24 | 71(13.87) | 26(29.21) | 0.002 |
| 25-44 | 342(66.80) | 51(57.30) |  |
| 45+ | 99(19.34) | 12(13.48) |  |
| **Marital Status** |  |  |  |
| Currently Married | 165(32.23) | 25(28.09) | 0.728 |
| Divorced/separated/widowed | 93(18.16) | 18(20.22) |  |
| Never married | 254(49.61) | 46(51.69) |  |
| **Occupation/Employment Status** |  |  |  |
| Employed | 305(59.57) | 48(53.93) | 0.351 |
| Unemployed | 207(40.43) | 41(46.07) |  |
| **Religion** |  |  |  |
| Christian | 355(65.43) | 53(59.55) | 0.283 |
| Others | 177(34.57) | 36(40. 45) |  |
| **Monthly Household Income** |  |  |  |
| <10,000 | 265(56.26) | 39(46.43) | 0.018 |
| 10,000-≤40,000 | 169(35.88) | 30(35.71) |  |
| >40,000 | 37(7.86) | 15(17.86) |  |
|  |  |  |  |
| **Family history of suicidality** |  |  |  |
| Yes | 111(21.68) | 36(40.45) | <0.001 |
| No | 401(78.32) | 53(59.55) |  |
|  |  |  |  |
| **Highest level of Education** |  |  |  |
| Level of education 0-8 | 188(36.72) | 35(39.33) | 0.544 |
| Level of education 8-13 | 210(41.02) | 39(43.82) |  |
| Level of education ≥14 | 114(22.27) | 15(16.85) |  |
| **Comorbid MDD** |  |  |  |
| Yes | 76(15.42) | 12(14.63) | 1.000 |
| No | 417(84.58) | 70(85.37) |  |
| **Comorbid Bipolar Disorder** |  |  |  |
| Yes | 321(65.11) | 58(70.73) | 0.379 |
| No | 172(34.89) | 24(29.27) |  |
| **Comorbid Substance use** |  |  |  |
| Yes | 320(62.50) | 70(78.65) | 0.004 |
| No | 192(37.50) | 19(21.35) |  |
| **Presence of chronic illness** |  |  |  |
| Yes | 270(52.84) | 54(60.67) | 0.205 |
| No | 241(47.16) | 35(39.33) |  |
| **Number of Life events** |  |  |  |
| 0-1 | 377(73.63) | 45(50.56) | <0.001 |
| 2-3 | 100(19.53) | 32(35.96) |  |
| 4 or more | 35(6.84) | 12(13.48) |  |
| **Negative symptoms** |  |  |  |
| Yes | 390(79.11) | 76(92.68) | 0.002 |
| No | 103(20.89) | 6(7.32) |  |
| **Symptoms of Delusion** |  |  |  |
| Yes | 358(69.92) | 61(68.54) | 0.803 |
| No | 154(30.08) | 28(31.46) |  |
| **Auditory Hallucinations** |  |  |  |
| Yes | 379(76.88) | 69(84.15) | 0.153 |
| No | 114(23.12) | 13(15.85) |  |

**Supplementary table 5: Variance inflation factor for testing multicollinearity**

| **Variable** | **VIF** | **1/VIF** |
| --- | --- | --- |
|  |  |  |
| **auditory hallucination** | **1.08** | **0.922537** |
| **negative life events** | **1.08** | **0.923106** |
| **substance use comorbidity** | **1.07** | **0.932778** |
| **bipolar disorder comorbidity** | **1.06** | **0.943776** |
| **symptoms of delusions** | **1.05** | **0.952134** |
| **negative symptoms** | **1.05** | **0.952487** |
| **age group** | **1.05** | **0.956503** |
| **employment status** | **1.04** | **0.958991** |
| **family history of suicidality** | **1.03** | **0.973644** |
|  |  |  |
| **Mean VIF** | **1.06** |  |
